# An observational study showed that explaining randomization using gambling-related metaphors and computer-agency descriptions impeded randomized clinical trial recruitment

**DOI:** 10.1016/j.jclinepi.2018.02.018

**Published:** 2018-07

**Authors:** Marcus Jepson, Daisy Elliott, Carmel Conefrey, Julia Wade, Leila Rooshenas, Caroline Wilson, David Beard, Jane M. Blazeby, Alison Birtle, Alison Halliday, Rob Stein, Jenny L. Donovan, CSAW study group, David Beard, Andrew Carr, Jonathan Cook, Cushla Cooper, Benjamin Dean, Jenny L. Donovan, Alastair Gray, Stephen Gwilym, Andrew Judge, Naomi Merritt, Jane Moser, Jonathan Rees, Ines Rombach, Julian Savulescu, Irene Tracey, Karolina Wartolowska, Chemorad study group, Jane M. Blazeby, Paul Barham, Sara T. Brookes, Tom Crosby, Jenny L. Donovan, Stephen J. Falk, S. Michael Griffin, William Hollingworth, Andrew D. Hollowood, Richard Krysztopik, Wyn Lewis, Jo Nicklin, Christopher Streets, Sean Strong, Dan Titcomb, Geraint Williams, POUT study group, Alison Birtle, Rik Bryan, James Catto, John Chester, Jenny L. Donovan, Ann French, Emma Hall, Chris Harris, Mark Johnson, Rob Jones, Francis Keeley, Tony Kirkbank, Roger Kockelbergh, Rebecca Lewis, Michelle Newton, Thomas Powles, Rachel Waters, Andrew Winterbottom, ACST-2 study group, Alison Halliday, Jean-Pierre Becquemin, Anna Belli, Marc Bosiers, Piergiorgio Cao, Christina Davies, Jenny L. Donovan, Alastair Gray, Michael Gough, Elizabeth Hayter, Peter Leopold, Sumaira McDonald, Jonathan Michaels, Borislava Mihaylova, Richard Peto, Steven Robertson, Peter Rothwell, Rachael Scott, Dafydd Thomas, Frank Vermassen, OPTIMA prelim study group, Rob Stein, John Bartlett, David Cameron, Amy Campbell, Peter Canney, Jenny L. Donovan, Janet Dunn, Helena Earl, Mary Falzon, Adele Francis, Peter Hall, Victoria Harmer, Helen Higgins, Louise Hiller, Luke Hughes-Davies, Claire Hulme, Iain Macpherson, Andreas Makris, Andrea Marshall, Christopher McCabe, Adrienne Morgan, Sarah Pinder, Christopher Poole, Daniel Rea, Nigel Stallard

**Affiliations:** a Population Health Sciences, University of Bristol, Canynge Hall, 39 Whatley Road, Bristol BS8 2PS, Bristol, United Kingdom; b Royal College of Surgeons Surgical Intervention Trials Unit (SITU), University of Oxford, Oxford, United Kingdom; c Rosemere Cancer Centre, Royal Preston Hospital, Sharoe Green Lane North, 12 Fulwood, Preston, Lancashire PR2 9HT4, United Kingdom; d Nuffield Department of Surgical Sciences, University of Oxford, Oxford OX3 9DU, United Kingdom; e University College London Hospitals (UCLH), Biomedical Research Centre (BMC), University College London Hospitals, 1st Floor Central, 250 Euston Road, London NW1 2PG, UK; f National Institute for Health Research Collaboration for Leadership in Applied Health Research and Care West (NIHR CLAHRC West), University Hospitals Bristol NHS Trust, Bristol, United Kingdom

**Keywords:** Randomized controlled trials, Recruitment, Randomization, Qualitative research, Recruitment to RCTs, Patient information

## Abstract

**Objectives:**

To explore how the concept of randomization is described by clinicians and understood by patients in randomized controlled trials (RCTs) and how it contributes to patient understanding and recruitment.

**Study Design and Setting:**

Qualitative analysis of 73 audio recordings of recruitment consultations from five, multicenter, UK-based RCTs with identified or anticipated recruitment difficulties.

**Results:**

One in 10 appointments did not include any mention of randomization. Most included a description of the method or process of allocation. Descriptions often made reference to gambling-related metaphors or similes, or referred to allocation by a computer. Where reference was made to a computer, some patients assumed that they would receive the treatment that was “best for them”. Descriptions of the rationale for randomization were rarely present and often only came about as a consequence of patients questioning the reason for a random allocation.

**Conclusions:**

The methods and processes of randomization were usually described by recruiters, but often without clarity, which could lead to patient misunderstanding. The rationale for randomization was rarely mentioned. Recruiters should avoid problematic gambling metaphors and illusions of agency in their explanations and instead focus on clearer descriptions of the rationale and method of randomization to ensure patients are better informed about randomization and RCT participation.

## Highlights

• Practices commonly used to describe randomisation in RCT recruitment could confuse patients.
• Patients found it difficult to comprehend gambling-related metaphors of randomisation.
• Computer-agency descriptions led to patients believing they would receive the best treatment.

**What is new?**

**Key findings**
• Recruiters found it difficult to explain randomization clearly to potential randomized clinical trial participants, using gambling-related metaphors to explain the element of chance or relying on implied agency in decision-making, for example, using a computer.
• Patients' responses were mostly indicative of lack of engagement but sometimes signaled discomfort and misunderstanding.
• Clear explanations of the rationale for randomization tended to occur only in response to patient-initiated requests.

**What this adds to what is known?**
• Clinical staff (doctors and nurses) need support and training to describe randomization.
• The use of gambling-related metaphors and computer agency in RCT discussion should be discouraged.

**What is the implication and what should change now?**
• Clinical staff (doctors and nurses) need support and training to describe randomization, and use of gambling-related metaphors and computer agency should be discouraged.

## 1 Introduction

Randomized controlled trials (RCTs) are the most rigorous study design to evaluate health-care interventions [1]. However, their success relies on patient recruitment, and this can be challenging [2]. Randomization or random allocation has been defined as: the process of assigning trial participants to treatment or control groups using an element of chance to determine the assignments to reduce bias (p.7) [3]. Research has indicated that the concept of randomization is difficult to communicate [4] and that patients can find it challenging to understand [5], [6]. Linked to this, it has been suggested that failure to accept randomization is a major reason for patients declining to participate in RCTs [7].

Guidelines for good clinical practice state that patients must be informed about the purpose of the trial, the treatment options, randomization, and the right to withdraw [8]. Guidance from the UK Health Research Authority (HRA) is available on how to describe randomization in patient information leaflets and recommends that the following points should be explained to patients: the reason for randomizing, that treatment will not be allocated in line with usual clinical decision-making, that treatment will be randomly allocated, and that neither the patient nor the doctor will decide the allocated treatment. In the guidance, it suggests that this process is “akin to drawing lots, tossing a coin, or rolling a die”, although specific details about the patient may be used to ensure groups in the trial are as similar as possible and that the patient is just as likely to receive either/any of the study arms [9].

Much of the research to date has reported on patients' difficulties with understanding randomization via self-reported questionnaires [10], or interview data completed post hoc, based on their responses to hypothetical scenarios [11], [12]. Relatively, little research has examined what recruiters actually say about randomization during recruitment appointments with some exceptions [13], [14], and patients' responses are even less commonly reported. The QuinteT Recruitment Intervention (QRI) [15] has demonstrated the benefit of investigating what is actually said during recruitment appointments [16], [17]. The QRI is an established recruitment intervention that includes a review and analysis of screening and recruitment data, interviews with recruiting clinicians, and audio recordings of consultations with patients where trial information is discussed. Thereafter, an action plan, typically in the form of support and specific training to help improve recruitment, is discussed and agreed with the Chief Investigator of the RCT. The aim of the QRI is to improve information delivery and increase participant recruitment and informed consent. This article is derived from the QRI research program and investigated how recruiters and patients discussed randomization in recruitment appointments. The findings illuminated the reasons why patients find the concept difficult to understand and identified opportunities for improvement. This article presents how randomization is communicated by health professionals and how patients respond to their descriptions, using data from five RCTs with actual or anticipated recruitment difficulties.

## 2 Method

### 2.1 Sampling

Data were taken from RCTs that included a QRI to support recruitment. For this analysis, data were available from five trials, all experiencing, or anticipated to have, recruitment difficulties. They included a wide range of specialisms (e.g., orthopedics, oncology, and general surgery), types of trials (e.g., surgery vs. nonsurgery vs. sham surgery, chemotherapy vs. surveillance, and two- or three-arm trials), and recruiters (surgeons, oncologists, research nurses [RNs], and physiotherapists). The analysis included all available recorded appointments from the five trials. The recordings were all made before the RCT receiving any feedback or training related to the recruitment intervention. Clinicians and patients were aware that the purpose of undertaking audio recording was to assist with trial recruitment and to improve information delivery. In total, 73 recruitment appointments, with 56 different patients and 27 different recruiters across five RCTs were audio-recorded. Recordings took place between 2010 and 2014. The QRI element of the studies was approved as part of the main trial Research Ethics Committee application in trials 1, 3, 4, and 5 and as a separate Research Ethics Committee application for trial 2.

Table 1 provides summary information of the participating trials and the range of recruiters providing information.

**Table 1:** RCT details and associated number of appointments recorded

RCT id	Clinical specialty	RCT comparison groups	Number of recordings (unique patients)	Appointment with		
				Doctor	Nurse	Joint appointment
1	Orthopedics	Group 1: Arthroscopy with surgical manipulation Group 2: Arthroscopy alone Group 3: Monitoring with specialist reassessment	16 (16)	12	4	0
2	Vascular medicine	Group 1: Surgical treatment Group 2: Stenting	8 (8)	5	3	0
3	Oncology	Group 1: Adjuvant treatment Group 2: Surveillance (with treatment offered on signs of cancer recurrence, if appropriate)	17 (13)	16	0	1
4	Oncology/surgery	Group 1: Neoadjuvant treatment and surgery Group 2: Definitive non-surgical treatment	16 (9)	11	0	5
5	Oncology	Group 1: Adjuvant treatment Group 2: Prognostic test-directed adjuvant treatment	16 (10)	7	2	7
		Total	73 (56 patients)	51	9	13

### 2.2 Data analysis

The qualitative analysis software package NVivo 10 (QSR international) was used to support data storage and analysis. M.J. listened to all of the recordings, following an approach of content analysis, and screened them to identify any discussion related to randomization. All references to randomization were extracted, transcribed, and coded. Documentation was also done where there was no reference to randomization. In keeping with Jenkins' analysis [18], we included explicit mentions of randomization, for example, where the word “randomization” or phrase “randomly allocated” was used as well as implicit mentions, for example, “you'll be allocated to either treatment x or treatment y”. D.E. and C.C. listened and independently coded a subset of 12 recordings. M.J., C.C., and D.E. met to compare coding and interpretation. Differing interpretations were discussed and resolved. The data presented in this article are transcribed excerpts from these consultations that provided an insight into what recruiters actually said to patients about randomization and also how patients responded. To preserve recruiters' anonymity, individual and trial identifiers have not been included. However, the excerpts presented are drawn from across all of the trials and were common in each trial.

## 3 Results

Information about randomization was presented to patients in various ways in the trials. In all cases, patients met at least two clinicians. In trials 1 and 2, information was first given to the patient by the relevant clinical specialist (often the principal investigator at the center) and thereafter further discussed with a RN in a separate encounter. In trial 3, patients had individual appointments with one specialist clinician and then with another. In trials 4 and 5, appointments were usually held with a clinician and RN present at the same time.

In three of the 73 recordings, the patient was identified as ineligible for the trial early in the appointment, making further discussion of the trial or randomization inappropriate; these recordings were excluded from further analysis. In seven of the remaining 70 consultations, there was no reference to randomization, with no clear reason for that information to be missing. None of the patients in these seven recordings chose to participate in their respective RCT.

Thematic analysis of the remaining 63 recordings revealed that there were three components to recruiters' randomization descriptions. Specifically, their descriptions covered the method of allocation, the process of allocation, and the reasons for randomization. Details of the trials and the number of consultations reviewed from each are shown in Table 1.

### 3.1 Method of allocation

Recruiters' first mention of any aspect of randomization often took the form of a description of the method of allocation (see Box 1). At its most basic level, this would simply be a statement of fact, without further explanation of how treatment would be allocated. This type of statement occurred in seven consultations. Recruiters regularly described the method of allocation in this manner although the effect of this was difficult to discern given the lack of response from patients in general. Typically, descriptions of the method of allocation followed presentation of details about the trial arms and included a brief mention of the percentage chance of receiving each trial treatment. The percentage chance of being allocated to one or another of the treatment arms was also referred to in trials with two arms. In some consultations, the concept of randomization was indicated to be “complex” or “quite complicated”.

Box 1 **Descriptions of the method of allocation**

Example 1.

• Research nurse: It's what we call a randomized control trial.
• Doctor: [Trial name] is a randomized study.

Example 2.

• Research nurse: Basically, randomisation means that one of those three things will happen so, in order for us to randomize you for the trial then you know that one of those options, you know, may happen and there is a 33% chance [of each].

Example 3.

• Doctor: Now, a few more things about [Trial 5]. It's a randomized study so that means that 50% of [people] who are in the study get the standard treatment, and 50% get [trial arm 2].

Example 4.

• Doctor: It's a fifty-fifty decision as to whether you get [treatment name] or not and that's not a decision we make, that's a, sort of through a randomisation process.

### 3.2 Process of allocation

We defined the process of allocation as the means by which patients would be allocated to trial treatment arms. Some discussion about the process of allocation featured in 33 consultations. There were two dominant, contrasting ways in which this was done. The most common (*n* = 19) was a description that implied there was some agency to how a decision would be made about treatment allocation. The majority of these (*n* = 15) recruiters stated that a computer would be involved in the decision-making process (see Box 2).

Box 2 **Descriptions of the process of allocation**

Example 1.

• Doctor: Essentially, we don't decide which treatment it is, but we let the computer decide because we're not sure whether one treatment is better than the other.
• Research nurse: It's done by a computer. They make the decision as to which one you do.

Example 2.

• Patient: Yeah, but putting in the machine, sometimes the machine can tell you better than what you can tell yourself.
• Relative: Well, like you say … the computer's going to come up with the best scenario for you so you can move from there.

Example 3.

• Doctor: So that's the situation that, that I want you to concentrate on is that there are two treatments you can go into … and the study will decide which you get.

Example 4.

• Doctor: And what we do is we take a large group of patients … and effectively toss a coin, we don't actually toss a coin, but effectively toss a coin, if it comes up heads you get [Treatment 1], if it comes up tails you don't.

Example 5.

• Research nurse: It's pot luck. A toss of a coin so to speak.

While it might be argued that what these recruiters were describing was broadly accurate, this approach could lead patients to believe that they would receive a form of treatment that was chosen as being “best for them” based on some or all of their personal characteristics, and evidence of this interpretation was found in patient responses:

• Doctor: If you were to choose to go into the trial we would then basically, they have a computer which, which chooses which treatment arm you go into and so, so there's that, that is exactly that.
• Patient: And is that going on like your symptoms of what you've had?

Immediately following this question, the recruiter attempted to clarify the apparent misunderstanding, explaining the “random” nature of the computer's decision:

• Doctor: half patients get it, half of them don't … there are a few factors they take into consideration to make that the, there are, you know, that, that there aren't equal numbers of certain types of patients but there's no, you know it's not chosen based on any particular characteristics. It is a random choice.

This misconception was not uncommon, and in six other consultations, patients, or their relatives, expressed a belief that the computer would be better at deciding what was best for them (see Box 2).

There was also evidence of recruiters apologizing for the use of a computer as part of the allocation process, suggesting discomfort on their part:

• RN: …that's [the random allocation] done by-it sounds terrible but by a computer system. But it's all set up by the statisticians.

In a further four consultations, the decision-making process was framed in a way that gave agency to the study or trial.

This approach generated the same misconceptions as those that gave agency to the computer. Shortly after the preceding extract, it was agreed that the patient should have some time to think about the decision and discuss it with family members and a RN. After a short exchange in which the RN stated that the patient was free to decline if she wished, the patient's response shows that she had misunderstood the “random” nature of allocation, and she believed that “the study” would choose the best treatment for her:

• Patient: …I would rather go into the study … [it]… knows which one is best for me.

In this instance, the RN responded to this misapprehension and immediately clarified that the RCT (or “study”) would not be picking what was best for the patient, but rather that the decision would be made by the “flick of a coin”. (RN).

This example introduces the second theme commonly observed in descriptions of the process of allocation; recruiters focusing on the “chance” element of how the allocation would be determined. In contrast to the previous articulation, this implied a lack of agency. These examples were less common but still featured in 14 of the 60 consultations that mentioned randomization.

The following examples typify how chance and the lack of agency were presented to patients potentially eligible for a two-arm trial:

• Doctor: So, the way it works is very simple. So, 50% of [patients] who join [trial name] at random are given [treatment 1]–toss of the coin.
• RN: …if you try and put it in simple terms it's literally the flick of a coin, on one side you've got [treatment 1], on the other side you've got [treatment 2]. We think they're equal so we can't make a choice so we go like that.

In both cases, the recruiter used a metaphor to describe the process of allocation, and in both cases, stated that there would be no consideration of patient factors in how the treatments were to be allocated. Yet, the process is not “literally” the flick of a coin, but rather certain factors will be taken into account to ensure patient groups in the two arms of the trial are comparable in terms of age or other key variables. A similar approach was also observed in several other consultations.

Across the data set, there were 15 instances of recruiters describing the chance nature of how allocation would be determined using these and other types of gambling metaphor, for instance allocation being likened to the “roll of a dice”, or patients being told to view allocation as something: “like a lottery”, or by having names drawn from a hat:

• Doctor: If you say that you were happy to go to trial at this point, you say, “I don't know which to go-I'll go into a trial.”—your name is drawn out of a hat as to which of those two options you will have.

Often patients did not say anything in response to these types of descriptions, other than to simply acknowledge what they had been told. However, where they did respond, these were all indicative of discomfort on their part. For example, in this short extract, a patient responded to a mention of a decision being made randomly in a startled manner:

• Doctor: We would just decide randomly.
• Patient: Oh crikey.

Presenting information in this way could lead to confusion and distress as well as discomfort:

• Patient: You wonder why. Why's all this happening? If it's a trial and it's a flip of a coin to which group err come on, y'know (laughing voice) this is my life.

This response is illuminating, in that it suggests that for this patient the use of a metaphor was problematic and perhaps even irreverent in the context. Ultimately, this patient declined to take part in the RCT. There was also evidence to suggest that recruiters anticipated that a random selection process would be undesirable to patients:

• Doctor: the only way to decide is to-pick a name out of a hat. And people do often think err-well they don't like it.

### 3.3 The reason for randomization

Discussions about the reason for a random allocation were notably lacking in all but 18 of the consultations.

In six of those 18, recruiters referred back to reasons why there was a randomized study, but this focused broadly on why the trial itself was taking place rather than on why randomization was necessary; recruiters spoke about there being a lack of available evidence for the RCT treatment(s) and made passing reference to random allocation in this context (see Box 3).

Box 3 **Descriptions of the reason for randomisation**

Example 1.

• Doctor: The trial that we're running is actually asking the question [about the best form of treatment] … and to answer that question what we need to do is take a large number or patients … and randomly allocate those patients into two groups.

Example 2.

• Doctor: The reason we do a randomized study [is] it's the only fair way of actually comparing two groups of patients.

Example 3.

• Research nurse: [this is] how actually most medical studies are done … with this random allocation … to make it a fair study.

Within these descriptions, there were occasional references to randomization being used as a means of ensuring the fairness of the process. These excerpts explained the reason for there being a randomized study but failed to make a connection to the need to compare outcomes across groups of people that were the same except for the treatment received.

In the remaining 12 consultations where the reason for randomization was described, recruiters described it as being a method of ensuring that there was no bias in the selection process. In each of these cases, it was described in a relatively succinct way, but there was no explanation of how randomization prevented bias or indeed of what bias meant in this context:

• RN: It [randomization] is done to make sure that it’s kind of an unbiased result.

It was interesting to note that although patient involvement in discussions about the method and process of allocation was minimal, discussions about the reason for randomization usually emerged as a consequence of a question from a patient or their relative. In response to such a question, the following recruiter provided an explanation that captured the rationale for randomization, which was then accepted by the patient:

• RN: Well, it's so that they have a group of [patients] where there's no way of there being bias or choosing in it. … They're comparing [people] who are otherwise exactly the same going into the trial.
• Patient: Yes I get it, I get that (RN).

In another consultation with the same recruiter, the patient's partner demonstrated their understanding of the explanation:

• Relative: So you're trying to take the bias out of this aren't you by having a random study.

In a further example, a patient responded to a description of the allocation being made by chance, by asking for clarification:

• RN: One of the conditions of the study is you have to be prepared to be randomly assigned to treatment.
• Patient: Why is that? Because if some people want [treatment 1] and some people [treatment 2] why aren't they put into the two separate pots and the remainder are dealt with randomly.

The patient had previously been told that treatment allocation was done “totally at random”. The recruiter subsequently explained that the only fair way to run a trial was for patients to be randomly allocated to treatment arms to avoid any bias:

• RN: We need to make sure that if there turns out to be a difference between those two different pots one way or the other … one pot does better or worse than the other one then we have to be sure that the reason for that difference is because we've done different things to the two different groups of people (RN).
…
• Patient: I can understand that logic better now.

The patient was satisfied with this explanation and consented to being randomized. However, explanations such as this were rarely present.

One recruiter had developed an approach of describing the reason for randomization which focused on the balance between the two groups of patients and used a similar form of words in each of the four recorded consultations:

• Doctor: [we have two groups of patients] …that should have the same number of men, the same number of women, the number [with worse disease] … of [less disease] etcetera, so the only difference between those two groups of patients is that one has had [treatment 1] and one group hasn't.

## 4 Discussion

In this article, we have explored how the process of random allocation is described and discussed in RCT recruitment appointments. Recruiters communicated two levels of difficulty in presenting information about randomization: first, an assumption that patients would dislike the concept of randomization as a means to determine treatment, and second that they would struggle to understand the meaning of a “randomized study” or the concept or purpose of randomization more specifically. They communicated their discomfort by stating that allocation was random that it was generally a complex process, or in some case, by not providing an explanation of randomization at all. When recruiters talked about the process of allocation, they often used metaphors related to chance (usually gambling) as part of their descriptions or referred to a computer or the RCT having agency over how allocation would be made. There was evidence from patient responses that these approaches were problematic and detrimental to patient understanding. We also noted that recruiters included the purpose or rationale for randomization much less often than the process and usually only in response to questions generated by patients.

Much of what is known empirically about how randomization is discussed is based on hypothetical situations [19], with members of the public who do not have the same level of investment in the information as they would if they were trial-eligible patients. Where the views of patients have been sought, they are not usually related to recruitment in a specific trial, rather just about generic information and overall understanding of randomization among the key concepts of RCTs [20]. In contrast, in this article, we demonstrated how, in practice, recruiters in five different RCTs went about explaining randomization to patients who were eligible for the respective RCTs and how patients responded to these explanations.

It has been suggested that the concept of randomisation is difficult to communicate [3]. Furthermore, patients can find the concept of randomisation to be challenging to understand [4], [21], [22] and that patients may not be prepared to be randomized to a trial if other options are available to them [23]. In our analysis, we saw recruiters describing the method of allocation in a relatively straightforward manner, usually referring to the percentage chance of a patient being allocated to one or other of the treatment arms. The approaches taken by many recruiters in describing this process of allocation, however, were often problematic. Recruiters commonly made references to a decision being made with some agency, with several making reference to the involvement of a computer or the study as part of the process of allocating treatment arms. The literature about descriptions of randomisation presents mixed views of the implications of referring to a computer in this context. For example, a focus group study reported patient anxiety about the de-personalization of the allocation process if undertaken by a computer [14]. In contrast, when members of the public and patients attending oncology outpatient clinics were asked to select their preferred description of the randomisation process from a list of seven alternatives [17], one of the most selected options was the following: A computer will randomly allocate you to one of two possible methods of treatment. Perhaps, the problem is not about reference to a computer per se but rather that patients are misinterpreting the role of the computer as providing some agency to the decision-making about their treatment allocation. This then creates an associated misunderstanding about the role of “the computer” in the decision-making process [24]. Lidz et al. [25] suggest that where patients were told that their treatment was decided by a computer, their “personal frame” led them to believe that they would receive a treatment designed for them. Given that patients have stated that they prefer it when their doctor makes decisions about their treatment allocation [26], it is likely that recruiters' reference to computer-aided decision-making provides a proxy for the doctor's expertise. Thus, perhaps unwittingly, by invoking a “computer” as the decision-making tool, recruiters are distanced from the potentially discomforting discussion with patients where they must acknowledge that there is uncertainty as to the best form of treatment. However, a problem with creating an impression that some decision-making agency exists is that it leads some patients to believe they will receive the best form of treatment for them—a therapeutic misconception [5], [27]. The problems associated with a therapeutic misconception include that patients end up with unrealistic optimism about the treatment they will receive being best fitted to their condition [28] as well as not being clearly informed about the justification for the trial. In the wider context, it perhaps reinforces the notion that patients may interpret the offer of trial participation as a “personal recommendation” from the clinician [29].

We observed recruiters using a range of metaphors to describe the chance nature of treatment allocation. Most of these were related to “gambling” in some way. For example, the roll of a dice, toss of a coin, or drawing lots. Metaphor has been previously reported as a component of recruiters' explanations [30] and often reported as being unpopular among patients. For example, gambling-related metaphors, such as drawing names from a hat, were disliked in focus group discussions with patients because of perceived associated risks to their lives [31]. Similarly, in an interview-based study with members of the public and staff and students at a medical school, the least preferred description of randomisation was reported to be one that referred to a coin being tossed [11], with some members of the public stating that they would not allow a treatment allocation to be made in that manner. However, where recordings of consultations had previously been coded, coin toss metaphors were one of the most commonly used phrases by clinicians [19]. This type of metaphor was also the most common in our data and identified as problematic by patients. Krieger [31] summarizes the problem of a coin toss metaphor as one which may lead patients to believe that they might “win” or “lose” in the randomisation process” according to the way the coin lands (2014: 1,171).

So, while metaphors may be a convenient way for recruiters to describe an unfamiliar concept (randomisation), linking it with a familiar concept associated with chance (gambling) appears to be flawed. The evidence in this article suggests that the use of such metaphors is still prevalent, and it remains recommended in the UK HRA guidance. These findings suggest that there is sufficient evidence to advise against the use of gambling-type metaphors.

Several studies report that the purpose of random allocation is poorly understood by patients [27], [32]. This article provides insight into why understanding may be poor: explanations of the purpose or rationale for randomisation are largely absent. Our findings align with those of Brown et al. [33]—in an observational study of oncology trial discussions, under half of 59 recordings of patient consultations featured information about the reason for randomisation. In our data, where such descriptions were present, they had often been initiated by patient questions rather offered by recruiters themselves. A clear implication of the rationale being either absent or poorly explained is that patients may not understand why they are being randomized. Managing patients' lack of understanding and/or acceptance of being randomly allocated to one or another treatment option adds to the emotional burden of discussing trials with patients [34]. Hence, offering clear explanations of why randomisation is happening could go some way to reducing that emotional burden, while also ensuring patients' decision-making about trial participation is better informed. We also found consultations where there was no mention of randomisation, and unsurprisingly, these patients were not recruited. In terms of improving trial recruitment, it has been reported that where more information is provided about the logic for a trial, patients who were previously uneasy about being randomized can change their mind [35]. It has been reported that training for those engaged in recruitment to RCTs can increase confidence in presenting concepts such as randomisation to patients [36], and this has been demonstrated in RCTs with embedded qualitative work [37].

## 5 Conclusions

This article provides a further demonstration of what qualitative research can bring to RCTs and their recruitment [38], [39], [40]. By examining the detail of what was said in actual consultations, we have been able to turn an empirical lens on concepts that to date have primarily been based on theoretical interpretations or post hoc analysis of the reported data. The way recruiters in these RCTs commonly tended to describe randomisation was often detrimental to patient recruitment and informed consent. There is clear evidence that recruiters should avoid problematic gambling metaphors and illusions of agency in their explanations and instead focus on clearer descriptions of the rationale and method of randomisation to enable patients to better understand this crucial part of the recruitment and informed consent process.

## Supplementary data

Data Profile
